# Critical periods and growth patterns from fetal life onwards associated with childhood insulin levels

**DOI:** 10.1007/s00125-016-4135-9

**Published:** 2016-10-18

**Authors:** Ellis Voerman, Vincent W. V. Jaddoe, Oscar H. Franco, Eric A. P. Steegers, Romy Gaillard

**Affiliations:** 1 The Generation R Study Group (Room Na-2915), Erasmus MC, University Medical Center, PO Box 2040, 3000 CA Rotterdam, the Netherlands; 2 Department of Epidemiology, Erasmus MC, University Medical Center, Rotterdam, the Netherlands; 3 Department of Pediatrics, Erasmus MC, University Medical Center, Rotterdam, the Netherlands; 4 Department of Obstetrics and Gynecology, Erasmus MC, University Medical Center, Rotterdam, the Netherlands

**Keywords:** Body mass index, Childhood, C-peptide, Fetal life, Growth, Insulin, Length, Prospective cohort

## Abstract

**Aims/hypothesis:**

We aimed to identify critical periods and specific longitudinal growth patterns from fetal life onwards associated with childhood insulin and C-peptide levels.

**Methods:**

In a prospective population-based cohort study of 4328 children, we repeatedly measured (femur) length and (estimated fetal) weight from the second trimester of fetal life until 6 years of age. BMI was calculated from 6 months onwards. Insulin and C-peptide levels were measured at 6 years of age.

**Results:**

Preterm birth and small or large size for gestational age at birth were not associated with childhood insulin levels. Conditional growth modelling showed that, independent of growth in other time intervals, weight growth in each time interval from birth onwards, length growth from 6 months onwards and BMI growth from 12 months onwards were positively associated with childhood insulin levels. The strongest associations were present for weight and BMI growth between 48 and 72 months of age. Repeated measurement analyses showed that, compared with children in the lowest quartile of childhood insulin, those in the highest quartile had a higher length from birth onwards and a higher weight and BMI from 24 months onwards. These differences increased with age. No associations were observed for fetal growth characteristics. Similar results were observed for C-peptide levels.

**Conclusions/interpretation:**

Our results suggest that rapid length, weight and BMI growth from birth onwards, but not during fetal life, is associated with higher insulin levels in childhood.

**Electronic supplementary material:**

The online version of this article (doi:10.1007/s00125-016-4135-9) contains peer-reviewed but unedited supplementary material, which is available to authorised users.

## Introduction

A large body of evidence suggests that adverse exposures in early life influence the risk of type 2 diabetes in later life [1]. Multiple previous studies have shown that adults born with either a low or high birthweight are at increased risk of type 2 diabetes [2, 3]. Birthweight is often used as a proxy for fetal growth. However, since birthweight is the result of different fetal growth patterns and is the starting point of infant growth, birthweight is not a causal factor per se in the development of type 2 diabetes. Studies assessing the associations of directly measured fetal growth characteristics with measures of glucose or insulin metabolism in later life are scarce and focused on measures of growth during specific trimesters only [4, 5]. As the development of the endocrine pancreas starts as early as the first trimester [6], fetal life might be a critical period for glucose and insulin metabolism, and the development of type 2 diabetes in later life.

More research has been performed on childhood growth patterns related to the risk of type 2 diabetes in later life [7–11]. These studies showed that participants who developed type 2 diabetes in adulthood were small at birth and thin in infancy. During childhood, they gained weight rapidly, leading to an average or above average BMI at the age of 11 years. Furthermore, weight gain during the first 6 months of life was more strongly related to the risk of insulin resistance in adulthood compared with weight gain later in infancy [9].

Thus far, there have been no studies to explore the associations of directly measured fetal growth characteristics or the combined associations of repeatedly measured fetal and childhood growth characteristics with insulin metabolism in later life. It is also not clear which time period of growth is most important for later insulin metabolism. Therefore, we aimed to identify critical periods and specific growth patterns from fetal life onwards that are important for the development of suboptimal insulin metabolism in childhood. Among 4328 participants of a population-based prospective cohort study from early pregnancy onwards, we assessed the associations of directly measured fetal and childhood growth characteristics with insulin and C-peptide levels at 6 years of age.

## Methods

### Study design

This study was embedded in the Generation R Study, a population-based prospective cohort study from early pregnancy onwards performed in Rotterdam, the Netherlands [12, 13]. The study was approved by the Medical Ethical Committee of the Erasmus Medical Center, University Medical Center, Rotterdam (MEC 198.782/2001/31). Written informed consent was obtained from all mothers. The response rate at birth was 61%. In total, 9901 children were enrolled in the study. As growth represents a change in size between two time points, we included children who had measurements of fetal or childhood growth characteristics available at, at least, two different time points (*n* = 9639). Of these children, 9395 were singleton and live-born, 6522 participated in the follow-up measurements at 6 years of age and 4328 had information on insulin or C-peptide levels available (ESM Fig. 1). Missing data on insulin and C-peptide levels were mainly due to lack of consent for venous punctures or non-successful venous punctures [13].

### Fetal and childhood growth characteristics

We performed fetal ultrasound examinations in each trimester of pregnancy. As described previously, gestational age was established using data from the first fetal ultrasound examination [14]. Second and third trimester fetal head circumference (HC), abdominal circumference (AC) and femur length (FL) were measured to the nearest millimetre using standardised ultrasound procedures. We calculated estimated fetal weight (EFW) using the formula of Hadlock et al [15]: log_10_ EFW = 1.5662 − 0.0108 (HC) + 0.0468 (AC) + 0.171 (FL) + 0.00034 (HC)^2^ − 0.003685 (AC × FL). Information on child’s sex, gestational age, weight and length at birth was obtained from medical records. Preterm birth was defined as a gestational age at birth <37 weeks. Low birthweight was defined as a birthweight ≤2500 g and high birthweight was defined as a birthweight ≥4000 g. Small and large size for gestational age at birth were defined as the lowest and the highest ten percentiles of gestational age and sex-adjusted birthweight in the complete cohort.

Well-trained staff in community health centres obtained childhood growth characteristics (length and weight) according to standard schedules and procedures at 6, 12, 24, 36 and 48 months of age. Growth characteristics at 72 months were obtained in a dedicated research centre following standardised protocols. We calculated BMI (kg/m^2^) at each time point from 6 months onwards. Standard deviation scores (SDS) were constructed for all growth characteristics using reference charts from the complete cohort for fetal measurements [14], northern European growth charts for birth measurements [16] and Dutch growth reference charts for childhood growth characteristics (Growth Analyzer 3.0; Dutch Growth Research Foundation, Rotterdam, the Netherlands) [17].

### Childhood insulin and C-peptide levels

At 6 years of age, 30 min fasting venous blood samples were obtained in a well-equipped and dedicated research centre in the Erasmus Medical Center Sophia Children’s hospital, Rotterdam, the Netherlands [12]. All blood samples were stored for a maximum of 4 h at 4°C. Blood samples were transported twice daily to the laboratory facility of the regional laboratory in Rotterdam, the Netherlands (STAR-MDC), where they were processed and stored within 4 h of venous puncture [13]. Insulin (pmol/l) and C-peptide levels (nmol/l) were obtained enzymatically using a Cobas 8000 Analyzer (Roche, Almere, the Netherlands). Quality control samples demonstrated intra- and inter-assay coefficients of variation of 1.39% and 2.40%, respectively. As C-peptide is secreted in equal amounts but has a longer half-life compared with insulin, we included C-peptide levels as an additional, more stable, outcome measure.

### Covariates

We assessed maternal age, pre-pregnancy weight, parity, ethnicity, educational level and folic acid use by questionnaire at enrolment in the study. Maternal height was measured and pre-pregnancy BMI was calculated. Smoking during pregnancy was repeatedly assessed by questionnaire throughout pregnancy. We obtained information on gestational hypertensive disorders (gestational hypertension and pre-eclampsia) and gestational diabetes from medical records [18]. Information on breastfeeding and the timing of introduction to solid foods was assessed by questionnaire during infancy, and the average time watching television was assessed by questionnaire at 6 years of age.

### Statistical analysis

First, we assessed the associations of gestational age at birth (preterm, term), birthweight (low, normal, high) and size for gestational age at birth (small, appropriate, large) with childhood insulin and C-peptide levels using multiple linear regression models. Second, to identify specific critical periods of growth associated with childhood insulin and C-peptide levels, we used conditional growth modelling [19–21]. We constructed length, weight and BMI growth variables, which are statistically independent from each other, using the standardised residuals obtained from the linear regression models of length, weight and BMI regressed on all prior corresponding growth measurements (ESM Methods 1). This enables inclusion of the growth measurements at all time points simultaneously in one model, and thus to estimate the independent and mutually adjusted influence of growth during each time interval on childhood insulin and C-peptide levels [19–21]. Third, in order to examine the associations of longitudinal fetal and childhood growth patterns with childhood insulin and C-peptide levels, we used unbalanced repeated measurement models. These models allow for incomplete outcome data and take the correlation between repeated measurements of the same participant into account by modelling the correlated errors of these measurements. For these analyses, we constructed quartiles of childhood insulin and C-peptide levels. These categories were included in the models as intercept and as interaction terms with (gestational) age to study the (gestational) age-independent effects (difference constant over time) as well as (gestational) age-dependent effects (difference non-constant over time). The actual models are described in more detail in ESM Methods 2.

All models were first adjusted for the child’s sex and age at insulin and C-peptide measurement only (basic models), and were subsequently adjusted for maternal and childhood sociodemographic and lifestyle related characteristics (adjusted models). The models for birth outcomes were also adjusted for childhood BMI at insulin and C-peptide measurement. We included covariates in the models based on their associations with the outcomes of interest in previous studies, a significant association with the determinants and outcomes, or a change in effect estimates of >10%. The associations between all growth characteristics and childhood insulin and C-peptide levels were linear. We tested for interactions between birthweight and BMI at the age of insulin and C-peptide measurement, birthweight and child’s sex, and BMI at the age of insulin and C-peptide measurement and child’s sex in the models described above, but no significant interactions were present (*p* values for interaction >0.05). In order to obtain normal distributions, we square root transformed insulin and C-peptide levels. SDS were constructed for insulin and C-peptide levels, defined as (observed value minus mean value of the reference population)/SD, to enable comparison of effect estimates. We used multiple imputation for missing values of covariates (<32%) and for the growth characteristics (<46%) for conditional growth modelling only by generating five independent datasets using the Markov Chain Monte Carlo (MCMC) method. Pooled effect estimates were presented. We performed a sensitivity analysis in children with growth characteristics available at all time points, which did not materially change the main findings (results not shown). The repeated measurement analyses were performed using the Statistical Analysis System version 9.3 (SAS Institute, Cary, NC, USA). All other analyses were performed using the Statistical Package of Social Sciences version 21.0 for Windows (IBM, Armonk, NY, USA).

## Results

### Study population

Table 1 shows the characteristics of the study population. At 6 years of age, the median insulin and C-peptide levels were 112 pmol/l (95% range 17, 395) and 0.95 nmol/l (95% range 0.30, 2.14), respectively. ESM Table 1 shows the growth characteristics at each time point. Non-response analysis showed that the mothers of children not included in the analyses were more likely to have a lower level of education and to smoke more often compared with the mothers of children included in the analyses (ESM Table 2). Furthermore, children not included in the analysis were less often from European descent. No or minor differences in childhood growth characteristics were observed between the groups.

**Table 1:** Characteristics of the study population

	Total group *N* = 4328
Maternal characteristics	
Age at intake (years), mean (SD)	30.7 (5.1)
Height (cm), mean (SD)	167.7 (7.5)
Pre-pregnancy weight (kg), median (95% range)	64.0 (49.0, 98.0)
Pre-pregnancy BMI (kg/m^2^), median (95% range)	22.7 (18.1, 34.5)
Highest education completed, *n* (%)	
Primary	343 (8.7)
Secondary	1692 (43.1)
Higher	1887 (48.1)
Parity, nulliparous (%)	2299 (55.1)
Ethnicity, European (%)	2605 (61.9)
Folic acid use, yes (%)	2239 (75.3)
Smoking during pregnancy, yes (%)	601 (16.0)
Gestational diabetes, yes (%)	42 (1.0)
Pre-eclampsia, yes (%)	65 (1.8)
Gestational hypertension, yes (%)	155 (4.2)
Child characteristics	
Sex, male (%)	2235 (51.6)
Gestational age at birth (weeks), median (95% range)	40.1 (35.8, 42.3)
Birthweight (g), median (95% range)	3450 (2261, 4474)
Ever breastfed, yes (%)	3150 (92.5)
Introduction of solid foods before 6 months, yes (%)	2328 (89.9)
Ethnicity, European (%)	2730 (64.8)
Television watching >2 h/day, *n* (%)	672 (19.9)
Age at 6-year follow-up examination (years), median (95% range)	6.0 (5.7, 8.0)
BMI (kg/m^2^), median (95% range)	15.9 (13.7, 21.2)
Insulin at 6 years (pmol/l), median (95% range)	112 (17, 395)
C-peptide at 6 years (nmol/l), median (95% range)	0.95 (0.30, 2.14)

### Critical periods of early growth

We did not observe associations of gestational age at birth, birthweight or size for gestational age at birth with childhood insulin levels in the basic or the adjusted models (Table 2). After additional adjustment for childhood current BMI, tendencies to inverse associations of birthweight and size for gestational age at birth with childhood insulin levels were present. The results were similar for childhood C-peptide levels (ESM Table 3).

**Table 2:** Associations of birth outcomes with childhood insulin levels (*n* = 4286)

Birth outcome	*n*	Insulin (SDS [95% CI])		
		Basic model	Adjusted model	BMI-adjusted model
Gestational age at birth				
Preterm (<37 weeks)	213	0 (−0.14, 0.14)	−0.03 (−0.18, 0.13)	−0.07 (−0.22, 0.08)
Term (≥37 weeks)	4051	Reference	Reference	Reference
*p* value for trend	4264	0.957	0.743	0.358
Birthweight				
Low (≤2500 g)	187	−0.01 (−0.16, 0.14)	0.06 (−0.12, 0.23)	0.12 (−0.06, 0.29)
Normal (2500–3999 g)	3474	Reference	Reference	Reference
High (≥4000 g)	625	−0.03 (−0.12, 0.05)	−0.07 (−0.16, 0.02)	−0.13 (−0.22, −0.04)*
*p* value for trend	4286	0.529	0.098	0.002
Size for gestational age				
SGA (≤10th percentile)	363	−0.01 (−0.11, 0.10)	0.01 (−0.10, 0.12)	0.08 (−0.03, 0.19)
AGA (10–90th percentile)	3447	Reference	Reference	Reference
LGA (≥90th percentile)	444	−0.04 (−0.14, 0.06)	−0.06 (−0.16, 0.04)	−0.12 (−0.22, −0.02)*
*p* value for trend	4254	0.635	0.276	0.005

Values are regression coefficients that reflect the differences in insulin SDS between the groups of the different birth outcomes

The basic models are adjusted for child’s sex and age at insulin and C-peptide measurement. The adjusted models are further adjusted for maternal pre-pregnancy BMI, maternal age, parity, smoking during pregnancy, folic acid use, maternal education level, gestational diabetes, gestational hypertensive disorders, ethnicity of the child, gestational age at birth (for birthweight) and birthweight (for gestational age at birth). The BMI-adjusted models are the adjusted models with additional adjustment for childhood BMI at insulin and C-peptide measurement

*p* values for trend were obtained by entering the categorical variables to the models as continuous variables

*p < 0.05

AGA, appropriate for gestational age; LGA, large for gestational age; SGA, small for gestational age

**Fig. 1:**
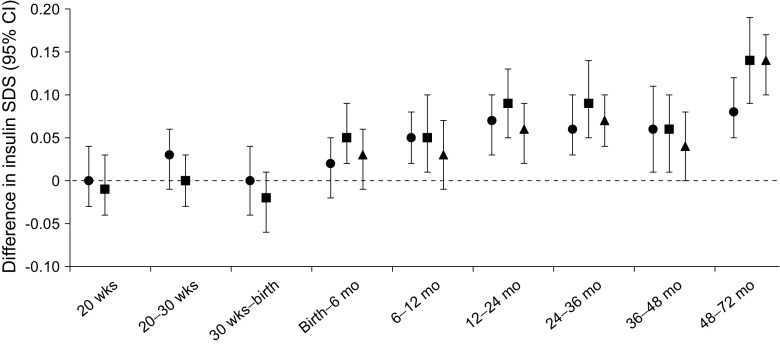
Associations of fetal and childhood growth, conditional on prior measurements, with childhood insulin levels (*n* = 4293). Values are regression coefficients representing differences in childhood insulin SDS per standardised residual change in growth characteristic in each time interval. The standardised residuals were obtained from models in which the growth measures of interest were regressed on the prior corresponding growth measures. For models presented for length and weight, the initial measure of size (starting point) was at 20 weeks of gestation (FL and EFW), and for BMI at 6 months of age. The models are adjusted for child’s sex, age at insulin and C-peptide measurement, maternal pre-pregnancy BMI, maternal age, parity, smoking during pregnancy, folic acid use, maternal education level, gestational diabetes, gestational hypertensive disorders, ethnicity of the child, gestational age at birth, breastfeeding, timing of introduction of solid foods and time watching television. Circles, length; squares, weight; triangles, BMI; wks, weeks; mo, months

Figure 1 shows the associations of growth during specific time intervals, conditional on prior growth measurements, with childhood insulin levels (adjusted models). No associations were observed for growth during specific periods in fetal life. Independent of growth in other time intervals, weight growth in each time interval from birth onwards, length growth in each time interval from 6 months onwards and BMI growth in each time interval from 12 months onwards were positively associated with childhood insulin levels (*p* < 0.05). The strongest associations were present for weight and BMI growth between 48 and 72 months. The results obtained from the basic model were consistent (ESM Fig. 2). Similar results were also observed for childhood C-peptide levels (ESM Figs 2, 3).

### Fetal and childhood growth patterns

**Fig. 2:**
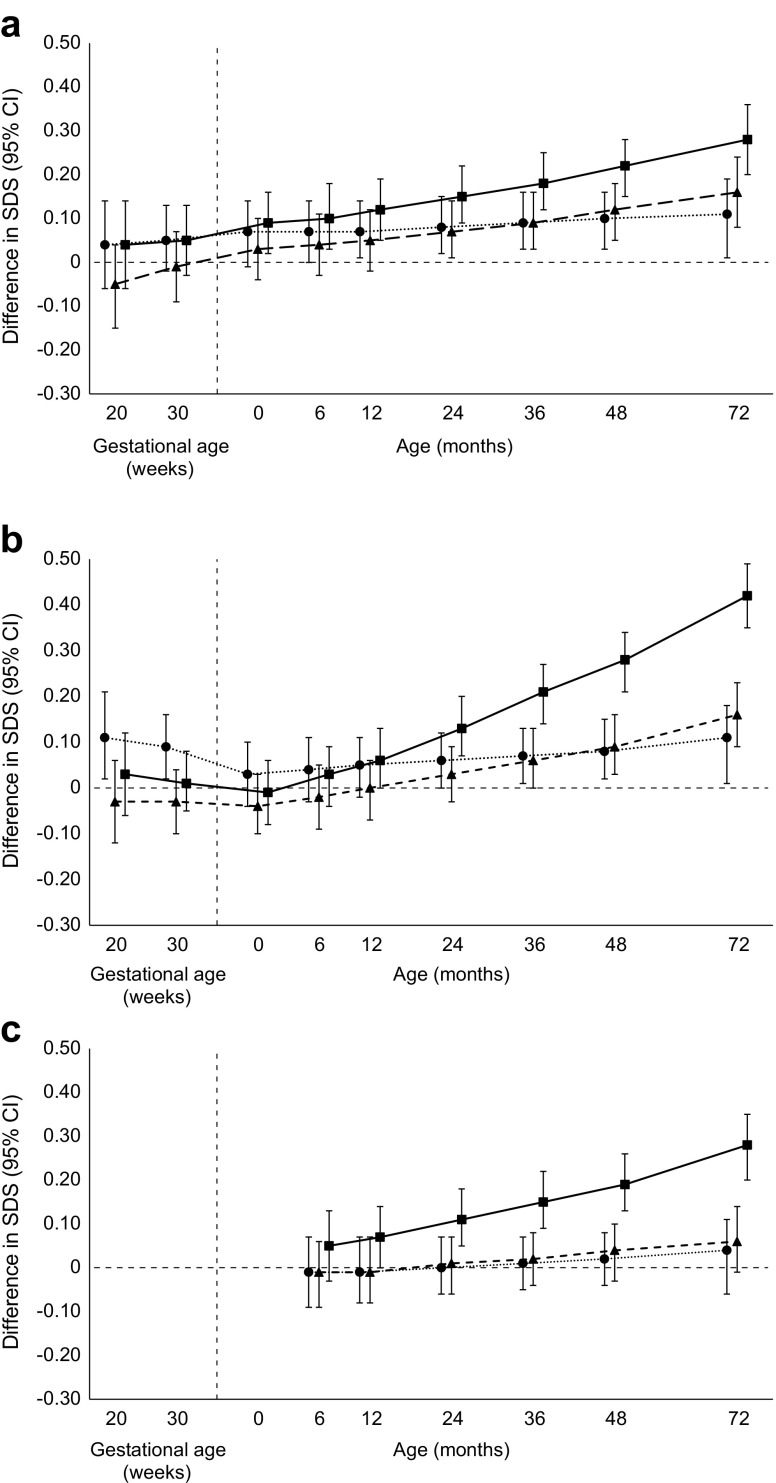
Fetal and childhood growth patterns according to insulin quartile (*n* = 4293). Results are based on repeated linear regression models and reflect the differences in SDS of (**a**) length (based on 39,022 measurements), (**b**) weight (based on 42,630 measurements) and (**c**) BMI (based on 23,277 measurements) growth in children with insulin levels in the second, third and fourth quartiles (insulin levels 62.7–112.4, 112.5–186.9 and 187.0–569.7 pmol/l, respectively), compared with those with insulin levels in the first quartile (2.7–62.6 pmol/l). The reference value is an SDS of 0. The models were adjusted for child’s sex, maternal pre-pregnancy BMI, maternal age, parity, smoking during pregnancy, folic acid use, maternal education level, gestational diabetes, gestational hypertensive disorders, ethnicity of the child, gestational age at birth, breastfeeding, timing of introduction of solid foods and time watching television. Circles/dotted line, second insulin quartile; triangles/dashed line, third insulin quartile; squares/solid line, fourth insulin quartile

Figure 2a–c shows the growth patterns from fetal life onwards for children in the higher childhood insulin quartiles compared with children in the lowest childhood insulin quartile. The overall growth patterns for fetal weight and length did not differ between the insulin quartiles (*p* values for trend >0.05). During childhood, length, weight and BMI gain were faster for children in the higher insulin quartiles (*p* values for trend <0.05). The largest differences were observed for children in the highest insulin quartile. Compared with children in the lowest insulin quartile, children in the highest insulin quartile were taller from birth onwards. Furthermore, they had a higher weight and BMI from 24 months onwards. These differences increased with age. ESM Fig. 4a–c shows the growth patterns associated with childhood C-peptide levels, which were similar to those for insulin levels. The regression coefficients for (gestational) age-independent (intercept) and (gestational) age-dependent effects (interaction between the insulin or C-peptide quartiles and (gestational) age) are given in ESM Tables 4 and 5.

## Discussion

In this prospective population-based cohort study, rapid length, weight and BMI growth from birth onwards was associated with higher childhood insulin levels. Although the strongest associations were present for weight and BMI growth between 48 and 72 months, we also observed independent associations of postnatal weight and BMI growth with childhood insulin levels at earlier time intervals. No associations were observed for fetal growth characteristics.

### Strengths and limitations

In this prospective population-based study, we had repeatedly measured growth characteristics available from fetal life onwards, enabling us to study the combined associations of fetal and childhood growth characteristics with childhood insulin and C-peptide levels. Of all eligible children at baseline, 54% were not included in the analyses. We consider it unlikely that this led to selection bias, since no or only minor differences were observed between the growth characteristics of children included in the analyses and children not included in the analyses.

The fasting time before venous puncture was limited. Due to the design of the study and the young age of the children, we were not able to collect blood samples after a longer fasting period [12]. This may have led to some non-differential misclassification and underestimation of the observed effect estimates. This may especially affect childhood insulin levels, which are less stable and have a shorter half-life compared with C-peptide levels. In addition, information on glucose levels was not available and therefore we were unable to estimate insulin sensitivity. However, as blood glucose levels are less variable than insulin levels, especially in children, and most of the variability in insulin sensitivity is due to insulin levels, we consider insulin levels to be a useful proxy of insulin sensitivity [22, 23]. Further studies are needed to assess the associations of fetal and childhood growth with detailed and fasting measures of offspring glucose and insulin metabolism.

As insulin is known to stimulate growth and fat deposition, it might be possible that the associations observed were due to reverse causation [24]. However, as information on insulin and C-peptide levels was only available at 6 years of age, we were not able to assess this possibility in our study.

The prospective design of the study from early pregnancy onwards enabled us to take into account numerous potential maternal and childhood confounders. However, some residual confounding might be present in the reported effect estimates as, for example, detailed dietary information was not available.

### Interpretation of main findings

An accumulating body of evidence suggests that adverse exposures in early life influence the risk of type 2 diabetes in later life. Multiple studies have reported associations of birthweight with measures of glucose and insulin metabolism in children as well as adults, some of them depending on adjustment for current body size [2–4, 9, 25]. We did not observe associations of gestational age at birth, birthweight or size for gestational age at birth with childhood insulin or C-peptide levels. After adjustment for childhood current BMI, tendencies to inverse associations for birthweight and size for gestational age at birth were present. However, it has been argued that adjusting early size for later size reflects the change in size between these two time points [26]. This suggests that the change between the growth characteristics at birth and in childhood is related to childhood insulin and C-peptide levels rather than birthweight per se.

Thus far, critical periods from fetal life onwards associated with the development of type 2 diabetes remain unclear, as previous studies mainly used birthweight as a proxy of fetal growth. Among 123 Danish adolescents, fetal growth velocity during the third trimester was not associated with any measure of insulin or glucose metabolism [4]. In our own study cohort, first trimester crown to rump length was not associated with childhood insulin levels [5]. In childhood, results from the Helsinki birth cohort suggest that growth during the first 6 months is critical for the development of insulin resistance in adulthood [9]. In a study of 1124 children from England and Wales, both a lower birthweight and a higher childhood ponderal index were associated with higher fasting and postload insulin levels at 10–11 years, with stronger effect estimates for the childhood ponderal index [25]. Furthermore, a recent study among 3301 European children with a mean age of 8.5 years showed that rapid BMI growth between 9 months and 6 years was related to a higher risk of insulin resistance [27]. In our current study, growth during fetal life was not associated with childhood insulin or C-peptide levels, independent of growth in other time intervals. Weight growth from birth onwards, length growth from 6 months onwards and BMI growth from 12 months onwards were independently and positively associated with childhood insulin and C-peptide levels. The strongest associations were present for weight and BMI growth between 48 and 72 months. These results suggest that growth during childhood, especially in weight and BMI, is important for the development of a suboptimal insulin metabolism in childhood, irrespective of growth during fetal life.

Observations from previous studies suggest that a growth pattern characterised by a low birthweight, followed by a rapid childhood weight gain, is associated with increased risk of insulin resistance or type 2 diabetes in later life [9–11]. We observed no associations of fetal growth patterns with childhood insulin and C-peptide levels. During childhood, children with higher insulin and C-peptide levels had a growth pattern characterised by a high weight and BMI, which increased throughout childhood. This suggests that children with relatively high childhood insulin or C-peptide levels grow faster during childhood but might not have grown differently during fetal life.

Thus, our results suggest that rapid growth throughout childhood is important for the development of suboptimal insulin metabolism in childhood, rather than fetal growth. It has been proposed that adverse exposures during fetal life lead to reduced muscle mass, reduced muscle sensitivity to insulin, and changes in the structure and function of the beta cells, which may subsequently lead to insulin resistance and beta cell dysfunction in later life [28–30]. Since only childhood growth characteristics were related to childhood insulin and C-peptide levels in our study, the mechanisms underlying these associations may involve differences in the body composition of the child, for example an increased body fat mass resulting from rapid postnatal growth or genetic influences, rather than developmental adaptations during fetal life [21, 31–33]. However, it is possible that associations of fetal growth with insulin or glucose metabolism become apparent later in life. Furthermore, the widely described associations of low birthweight with increased risk of type 2 diabetes may be explained by beta cell dysfunction, which we were unable to assess in this study [34]. If our findings are confirmed by other studies with directly measured fetal growth data, detailed childhood growth data and more detailed measures of childhood glucose and insulin metabolism, including measures of beta cell function, our results underline the importance of strategies aimed at preventing rapid weight gain throughout childhood to improve insulin metabolism in later life.

## Conclusions

Our results suggest that rapid length, weight and BMI gain from birth onwards, but not growth during fetal life, is associated with the development of suboptimal insulin metabolism in childhood. Further research is needed to replicate our findings and to explore the underlying mechanisms.

## Electronic supplementary material

Below is the link to the electronic supplementary material.

ESM (PDF 450 kb)

## Abbreviations

AC: Abdominal circumference
AGA: Appropriate for gestational age
EFW: Estimated fetal weight
FL: Femur length
HC: Head circumference
LGA: Large for gestational age
SDS: Standard deviation score
SGA: Small for gestational age
