# Supplementary material for: Critical periods and growth patterns from fetal life onwards associated with childhood insulin levels
Source: Diabetologia. 2016 Oct 18;60(1):81–8. doi: 10.1007/s00125-016-4135-9 (PMC5495163; doi:10.1007/s00125-016-4135-9)
Supplement: Supplementary file 1 — (PDF 450 kb) [file 125_2016_4135_MOESM1_ESM.pdf]

Electronic supplementary material

Critical periods and growth patterns from fetal life onwards associated with childhood insulin levels

Ellis Voerman[1,2,3], Vincent W.V. Jaddoe[1,2,3], Oscar H. Franco[2], Eric A. P. Steegers[4], Romy Gaillard[1,2,3]

[1]The Generation R Study Group (Room Na-2915), Erasmus MC, University Medical Center, PO Box 2040, 3000 CA Rotterdam, the Netherlands

[2]Department of Epidemiology, Erasmus MC, University Medical Center, Rotterdam, the Netherlands

[3]Department of Pediatrics, Erasmus MC, University Medical Center, Rotterdam, the Netherlands

[4]Department of Obstetrics and Gynecology, Erasmus MC, University Medical Center, Rotterdam, the Netherlands

ESM Methods 1 Conditional growth modelling

We used conditional growth modelling in order to identify critical periods of growth associated with childhood insulin and C-peptide levels [1-3]. We first calculated the predicted value of the growth measurement at the time point of interest, using a linear regression model including all prior corresponding growth measurements. The standardised residuals of these models indicate to what degree a growth measure of interest differs from that predicted by all previous growth measures, and represent the excess growth during the time interval prior to the growth measurement of interest. As these standardised residuals are not correlated, we subsequently included the standardised residuals plus the initial measure of size (femur length and estimated fetal weight at 20 weeks of gestation for models of length and weight, and BMI at the age of 6 months for models of BMI) simultaneously in one linear regression model, estimating the effect of growth during each time interval on childhood insulin or C-peptide levels. The regression coefficients of these models represent the difference in childhood insulin or C-peptide levels per standardised residual change in growth in each time interval, adjusted for growth during the other time intervals. Conditional growth modelling enables us to examine the associations of growth during specific time-periods, independent of growth in other time intervals and to compare the strength of the effect estimates between time intervals. This method thus allows us to identify periods of growth which are most important for later childhood insulin and C-peptide levels [1-3].

References

1. Keijzer-Veen MG, Euser AM, van Montfoort N, Dekker FW, Vandenbroucke JP, Van Houwelingen HC (2005) A regression model with unexplained residuals was preferred in

the analysis of the fetal origins of adult diseases hypothesis. J Clin Epidemiol 58:1320-1324

2. Jones A, Charakida M, Falaschetti E, Hingorani AD, Finer N, Masi S, et al (2012) Adipose and height growth through childhood and blood pressure status in a large prospective cohort study. Hypertension 59:919-925

3. Gishti O, Gaillard R, Manniesing R, et al (2014) Fetal and infant growth patterns associated with total and abdominal fat distribution in school-age children. J Clin Endocrinol Metab 99:2557-2566

ESM Methods 2 Unbalanced repeated measurement models

We analysed the fetal and childhood growth patterns among children in the 2nd, 3rd and 4th insulin and C-peptide quartiles, as compared to children in the 1st quartiles using unbalanced repeated measurement regression models. These models allow for incomplete outcome data and take the correlation between repeated measurements of the same participant into account by modelling the correlated errors of these measurements [1, 2]. As fetal and childhood growth were defined using different measures (femur length vs. body length and estimated fetal weight vs. measured weight), we constructed best fitting models for fetal and childhood growth separately. First, we constructed linear models for fetal and childhood growth characteristics separately and subsequently tested whether adding higher polynomials of (gestational) age to the models improved the fit of the models by checking the goodness of fit (smallest –2 log likelihood). Since higher polynomials of (gestational) age did not considerably improve the fit of the models and to prevent overfitting of the models, we kept the models as simple as possible and did not include higher polynomials of (gestational) age to the models. To model the correlated errors, a compound symmetry covariance structure was assumed for the models for fetal growth, indicating that measurements are correlated equally. For the childhood models, we assumed an autoregressive covariance structure, given that measurements closer in time tend to be correlated more strongly. Using alternative covariance structures did not change the results.

The final models can be written as:

Fetal length (SDS) = ß0 + ß1 × insulin or C-peptide quartile + ß2 × gestational age + ß3 × insulin or C-peptide quartile × gestational age

Childhood length (SDS) = ß0 + ß1 × insulin or C-peptide quartile + ß2 × age + ß3 × insulin or C-peptide quartile × age

Fetal weight (SDS) = ß0 + ß1 × insulin or C-peptide quartile + ß2 × gestational age + ß3 × insulin or C-peptide quartile × gestational age

Childhood weight (SDS) = ß0 + ß1 × insulin or C-peptide quartile + ß2 × age + ß3 × insulin or C-peptide quartile × age

BMI (SDS)= ß0 + ß1 × insulin or C-peptide quartile + ß2 × age + ß3 × insulin or C-peptide quartile × age

In these models, ‘ß0 + ß1 × insulin or C-peptide quartile’ reflects the intercept. The intercept reflects the mean growth characteristic value in SDS for each insulin or C-peptide quartile. The term ‘ß2 × (gestational) age’ reflects the change in growth characteristics per week (fetal models) or month (childhood models). The term ‘ß3 × insulin or C-peptide quartile × (gestational) age’, reflects the difference in change in growth characteristics per week (fetal models) or month (childhood models) between the different insulin or C-peptide quartiles. For presentation purposes, we obtained point estimates from these models at time points of interest. The regression coefficients for (gestational) age-independent (intercept: ß0 + ß1 × insulin or C-peptide quartile) and (gestational) age-dependent differences (slope: ß3 × insulin or C-peptide quartile × (gestational) age) are given in ESM Tables 4 and 5 below.

References

1. Goldstein H (1995) Multilevel Statistical Methods. 2nd edn. Edward Arnold, London

2. Royston P, Ambler G, Sauerbrei W (1999) The use of fractional polynomials to model continuous risk variables in epidemiology. Int J Epidemiol 28:964–74

ESM Table 1 Fetal and childhood growth characteristics

	Total group n=4328
**Second trimester**	
Gestational age (weeks), median (95% range)	20.5 (18.6, 23.3)
Femur length (mm), mean (SD)	34 (4)
Estimated fetal weight (g), median (95% range)	364 (246, 624)
**Third trimester**	
Gestational age (weeks), median (95% range)	30.4 (28.4, 33.1)
Femur length (mm), mean (SD)	58 (3)
Estimated fetal weight (g), median (95% range)	1608 (1175, 2232)
**Birth**	
Gestational age at birth (weeks), median (95% range)	40.1 (35.8, 42.3)
Birth length (cm), mean (SD)	50.2 (2.3)
Birthweight (g), median (95% range)	3450 (2261, 4474)
**6 months**	
Age at follow-up (months), median (95% range)	6.2 (5.2, 8.3)
Length (cm), mean (SD)	67.7 (2.6)
Weight (kg), mean (SD)	7.9 (0.9)
BMI (kg/m^2), mean (SD)	17.2 (1.4)
**12 months**	
Age at follow-up (months), median (95% range)	11.1 (10.1, 125)
Length (cm), mean (SD)	74.4 (2.6)
Weight (kg), mean (SD)	9.7 (1.1)
BMI (kg/m^2), mean (SD)	17.4 (1.4)
**24 months**	
Age at follow-up (months), median (95% range)	11.1 (10.1, 12.5)
Height (cm), mean (SD)	88.8 (3.7)
Weight (kg), mean (SD)	13.0 (1.6)
BMI (kg/m^2), median (95% range)	16.5 (14.1, 19.6)
**36 months**	
Age at follow-up (months), median (95% range)	37.7 (35.3, 40.7)
Height (cm), mean (SD)	97.5 (3.8)
Weight (kg), mean (SD)	15.3 (1.9)
BMI (kg/m^2), mean (SD)	16.0 (1.3)
**48 months**	
Age at follow-up (months), median (95% range)	45.7 (44.4, 48.4)
Height (cm), mean (SD)	103.3 (4.1)
Weight (kg), mean (SD)	17.0 (2.2)

BMI (kg/m^2), mean (SD)	15.8 (1.4)
**72 months**	
Age at follow-up (months), median (95% range)	72.6 (68.2, 96.3)
Height (cm), mean (SD), cm	119.7 (6.1)
Weight (kg), median (95% range)	22.6 (17.6, 34.4)
BMI (kg/m^2), median (95% range)	15.9 (13.7, 21.2)

ESM Table 2 Non-response analysis of children included and not included in the analyses (n=9395)

	Included in the analyses<br>n=4328	Not included in the analyses<br>n=5067	p-value[a]
**Maternal characteristics**			
Age at intake (years), mean (SD)	30.7 (5.1)	29.2 (5.5)	<0.001
Height (SD), mean (SD)	167.7 (7.5)	166.6 (7.3)	<0.001
Pre-pregnancy weight (kg), median (95% range)	64.0 (49.0, 98.0)	63.0 (47.0, 100.0)	0.039
Pre-pregnancy BMI (kg/m^2), median (95% range)	22.7 (18.1, 34.5)	22.6 (17.8, 35.5)	0.684
Highest education completed, n (%)			
Primary	343 (8.7)	565 (13.0)	<0.001
Secondary	1692 (43.1)	2117 (48.7)	
Higher	1887 (48.1)	1662 (38.3)	
Parity, Nulliparous (%)	2299 (55.1)	2709 (55.4)	0.747
Ethnicity, European (%)	2605 (61.9)	2502 (54.5)	<0.001
Folic acid use, yes (%)	2239 (75.3)	2252 (66.5)	<0.001
Smoking during pregnancy, yes (%)	601 (16.0)	837 (19.8)	<0.001
Gestational diabetes, yes (%)	42 (1.0)	57 (1.2)	0.444
Pre-eclampsia, yes (%)	65 (1.8)	103 (2.4)	0.060
Gestational hypertension, yes (%)	155 (4.2)	155 (3.6)	0.167
**Child characteristics**			
Sex, male (%)	2235 (51.6)	2522 (49.8)	0.074
Gestational age at birth (weeks), median (95% range)	40.1 (35.8, 42.3)	39.8 (35.3, 42.3)	0.038
Birthweight (g), median (95% range)	3450 (2261, 4474)	3400 (2190, 4510)	<0.001
Ever breastfed, yes (%)	3150 (92.5)	2919 (91.4)	0.086
Timing of introduction of solid foods, No. before 6 months (%)	2328 (89.9)	1941 (89.0)	0.300
Ethnicity, European (%)	2730 (64.8)	2686 (58.3)	<0.001
Age at follow-up examination at 24 months (months), median (95% range)	25.0 (23.4, 31.4)	25.0 (23.4, 31.3)	0.815
BMI at the age of 24 months (kg/m^2), mean (SD)	16.6 (1.4)	16.6 (1.5)	0.590
Age at follow-up examination at 48 months (months), median (95% range)	45,8 (44.4, 48.4)	45.8 (44.5, 48.7)	0.004
BMI at the age of 48 months, mean (SD), kg/m^2	15.8 (1.4)	15.9 (1.5)	0.033
Age at follow-up examination at 6 years (years), median (95% range)	6.0 (5.7, 8.0)	6.0 (5.6, 7.6)	<0.001

BMI at the age of 6 years (kg/m^2), mean (SD)	16.2 (1.8)	16.2 (2.0)	0.651

[a]Differences in participant characteristics between the groups were tested using Independent Samples T-tests for continuous variables and Chi-square tests for proportions.

ESM Table 3 Associations of birth outcomes with childhood C-peptide levels (n=4321)

		C-peptide (SDS (95% CI))		
**Birth outcome**	**n**	**Basic model**	**Adjusted model**	**BMI adjusted model**
**Gestational age at birth**				
Preterm (<37 weeks)	215	0.03 (-0.11, 0.17)	0 (-0.15, 0.16)	-0.04 (-0.19, 0.12)
Term (≥ 37 weeks)	4083	Reference	Reference	Reference
p-value for trend	4298	0.683	0.975	0.647
**Birthweight**				
Low (≤2500 g)	191	0.09 (-0.06, 0.24)	0.15 (-0.03, 0.32)	0.20 (0.03, 0.37)*
Normal (2500-3999 g)	3503	Reference	Reference	Reference
High (≥4000 g)	627	0 (-0.09, 0.08)	-0.05 (-0.14, 0.04)	-0.10 (-0.19, -0.01)*
p-value for trend	4321	0.465	0.095	0.005
**Size for gestational age**				
SGA (≤ 10th percentile)	368	0.03 (-0.08, 0.14)	0.05 (-0.06, 0.16)	0.10 (-0.01, 0.21)
AGA (10 - 90th percentile)	3475	Reference	Reference	Reference
LGA (≥90th percentile)	445	0 (-0.10, 0.10)	-0.03 (-0.13, 0.07)	-0.08 (-0.18, 0.02)
p-value for trend	4288	0.730	0.236	0.011

Values are regression coefficients that reflect the differences in C-peptide SDS between the groups of the different birth outcomes. The basic models are adjusted for child's sex and age at insulin and C-peptide measurement. The adjusted models are further adjusted for maternal pre-pregnancy BMI, maternal age, parity, smoking during pregnancy, folic acid use, maternal education level, gestational diabetes, gestational hypertensive disorders, ethnicity of the child, gestational age at birth (for birthweight) and birthweight (for gestational age at birth). The BMI-adjusted models are the adjusted models with additional adjustment for childhood BMI at insulin and C-peptide measurement. P-values for trend were obtained by entering the categorical variables to the models as continuous variables. *p <0.05. AGA, Appropriate for gestational age. LGA, Large for gestational age; SGA: Small for gestational age

ESM Table 4 Longitudinal associations between insulin levels and growth characteristics (n=4293)

Childhood insulin levels	Intercept (SDS)	p-value	Slope (SDS (95% CI))	p-value
**Height**				
**Fetal**				
1st insulin quartile	-0.287	0.27	Reference	
2nd insulin quartile	-0.273	0.90	0.0013 (-0.0059, 0.0086)	0.72
3rd insulin quartile	-0.437	0.18	0.0048 (-0.0024, 0.0119)	0.19
4th insulin quartile	-0.273	0.90	0.0012 (-0.0060, 0.0084)	0.74
**Childhood**				
1st insulin quartile	-4.426	<0.001	Reference	
2nd insulin quartile	-4.358	0.07	0.0005 (-0.0004, 0.0017)	0.24
3rd insulin quartile	-4.395	0.41	0.0014 (0.0007, 0.0028)	<0.001
4th insulin quartile	-4.337	0.02	0.0022 (0.0016, 0.0037)	<0.001
**Weight**				
**Fetal**				
1st insulin quartile	-0.508	0.02	Reference	
2nd insulin quartile	-0.345	0.08	-0.0024 (-0.0080, 0.0031)	0.38
3rd insulin quartile	-0.547	0.68	0.0003 (-0.0052, 0.0058)	0.90
4th insulin quartile	-0.440	0.46	-0.0018 (-0.0073, 0.0037)	0.52
**Childhood**				
1st insulin quartile	-3.455	<0.001	Reference	
2nd insulin quartile	-3.421	0.34	0.0010 (0.0001, 0.0020)	0.03
3rd insulin quartile	-3.490	0.32	0.0027 (0.0017, 0.0037)	<0.001
4th insulin quartile	-3.463	0.78	0.0060 (0.0050, 0.0068)	<0.001
**BMI**				
1st insulin quartile	-1.821	<0.001	Reference	
2nd insulin quartile	-1.865	0.71	0.0008 (-0.0006, 0.0021)	0.26
3rd insulin quartile	-1.871	0.62	0.0012 (-0.0002, 0.0251)	0.08
4th insulin quartile	-1.865	0.50	0.0035 (0.0021, 0.0048)	<0.001

Values are regression coefficients obtained from linear repeated measurement models and reflect the (gestational) age independent differences (intercepts) and the (gestational) age dependent differences (slopes: change in growth characteristics SDS per week (fetal models) or per month (childhood models) per insulin quartile, compared with the reference group (1st insulin quartile). The models were adjusted for child's sex, maternal pre-pregnancy BMI, maternal age, parity, smoking during pregnancy, folic acid use, maternal education level, gestational diabetes, gestational hypertensive disorders, ethnicity of the child, gestational age at birth, breastfeeding, timing of introduction of solid foods and time watching television. P-values reflect the significance levels of the regression coefficients

ESM Table 5 Longitudinal associations between C-peptide levels and growth characteristics (n=4303)

Childhood C-peptide levels	Intercept (SDS)	p-value	Slope (SDS (95% CI))	p-value
**Height**				
**Fetal**				
1st C-peptide quartile	-0.279	0.29	Reference	
2nd C-peptide quartile	-0.220	0.60	-0.0012 (-0.0085, 0.0059)	0.73
3rd C-peptide quartile	-0.394	0.30	0.0046 (-0.0026, 0.0118)	0.21
4th C-peptide quartile	-0.398	0.29	0.0052 (-0.0021, 0.0125)	0.16
**Childhood**				
1st C-peptide quartile	-4.406	<0.001	Reference	
2nd C-peptide quartile	-4.383	0.54	0.0018 (0.0007, 0.0028)	<0.001
3rd C-peptide quartile	-4.375	0.42	0.0016 (0.0006, 0.0027)	<0.01
4th C-peptide quartile	-4.406	0.32	0.0032 (0.0021, 0.0042)	<0.001
**Weight**				
**Fetal**				
1st C-peptide quartile	-0.562	0.01	Reference	
2nd C-peptide quartile	-0.375	0.05	-0.0065 (-0.0120, -0.0010)	0.02
3rd C-peptide quartile	-0.556	0.95	-0.0006 (-0.0062, 0.0049)	0.83
4th C-peptide quartile	-0.554	0.93	-0.0009 (-0.0065, 0.0047)	0.75
**Childhood**				
1st C-peptide quartile	-3.440	<0.001	Reference	
2nd C-peptide quartile	-3.482	0.24	0.0026 (0.0017, 0.0035)	<0.001
3rd C-peptide quartile	-3.486	0.20	0.0033 (0.0024, 0.0043)	<0.001
4th C-peptide quartile	-3.482	0.25	0.0057 (0.0048, 0.0067)	<0.001
**BMI**				
1st C-peptide quartile	-1.872	<0.001	Reference	
2nd C-peptide quartile	-1.872	0.92	0.0011 (-0.0002, 0.0024)	0.10
3rd C-peptide quartile	-1.897	0.56	0.0019 (0.0006, 0.0033)	<0.01
4th C-peptide quartile	-1.852	0.63	0.0033 (0.0020, 0.0047)	<0.001

Values are regression coefficients obtained from linear repeated measurement models and reflect the (gestational) age independent effects (intercepts) and the (gestational) age dependent effects (slopes, change in growth characteristics SDS per week (fetal models) or per month (childhood models) per C-peptide quartile, compared with the reference group (1st C-peptide quartile). The models were adjusted for child's sex, maternal pre-pregnancy BMI, maternal age, parity, smoking during pregnancy, folic acid use, maternal education level, gestational diabetes, gestational hypertensive disorders, ethnicity of the child, gestational age at birth, breastfeeding, timing of introduction of solid foods and time watching television. P-values reflect the significance levels of the regression coefficients.

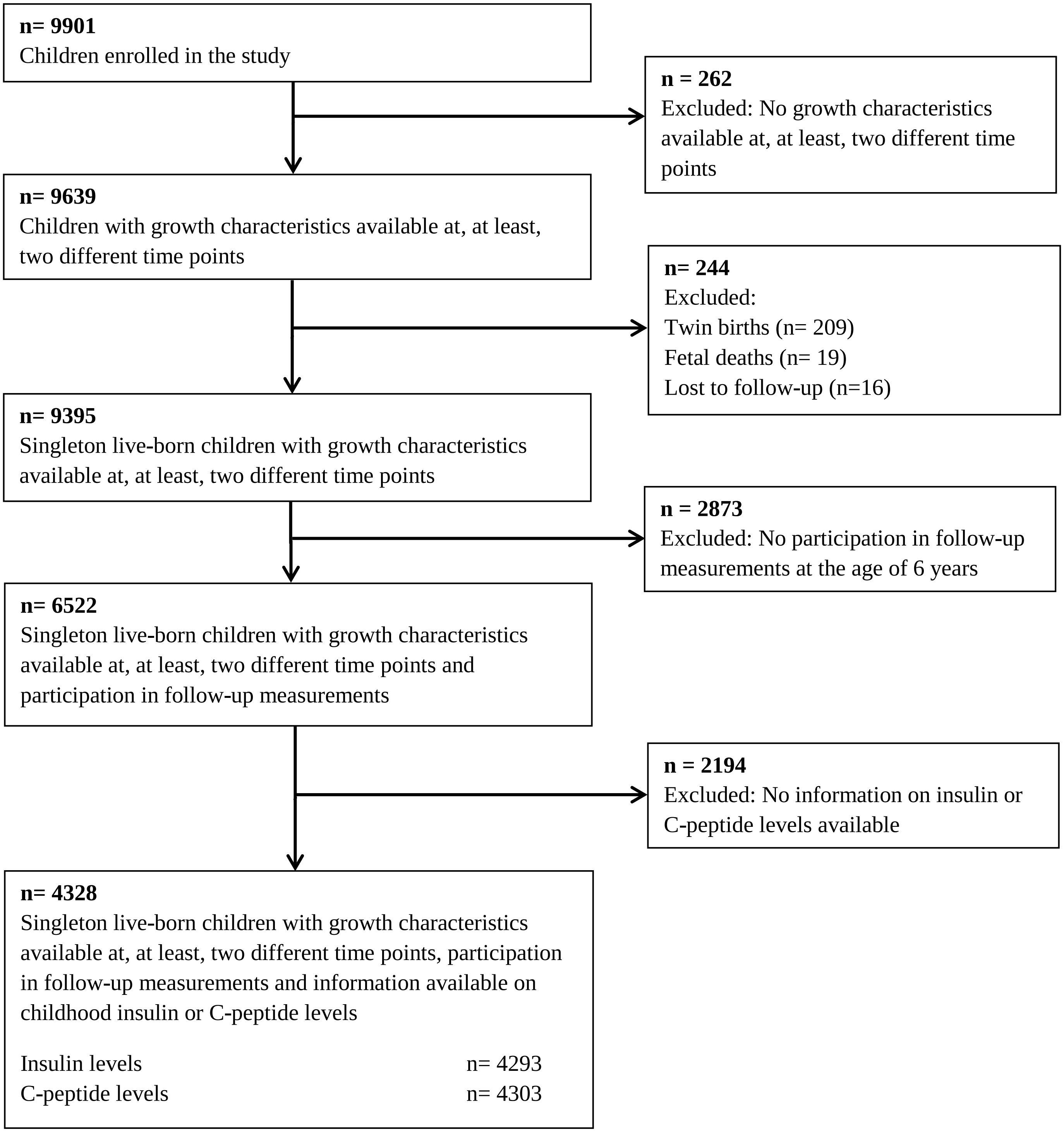

ESM Fig. 1 Flow chart of the study participants

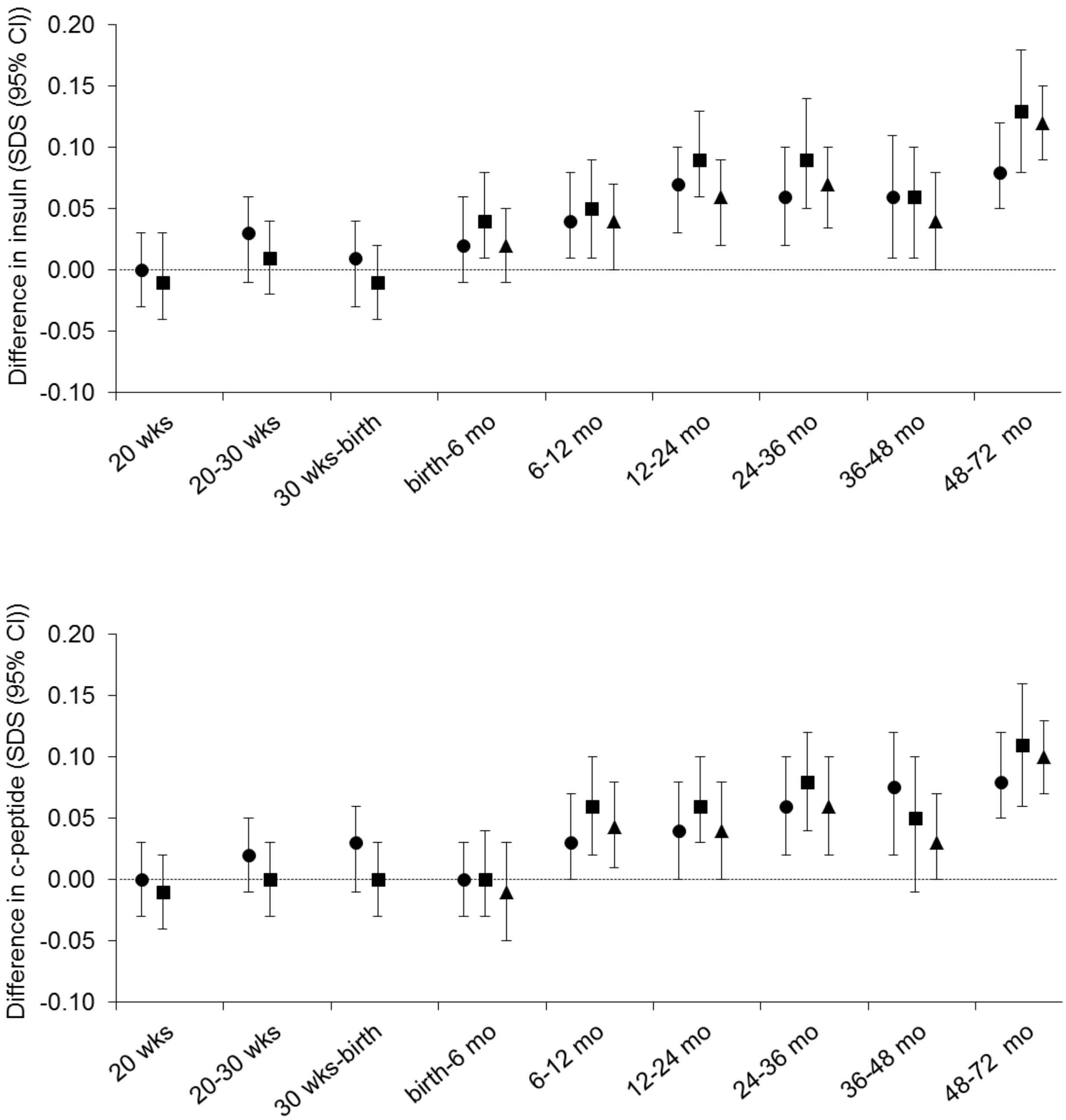

ESM Fig. 2 Associations of fetal and childhood growth conditional on prior measurements with childhood insulin and C-peptide levels (n=4328). Values are regression coefficients representing differences in childhood insulin or C-peptide SDS per standardised residual change in growth characteristic in each time interval. The standardised residuals were obtained from models in which the growth measures of interest were regressed on the prior corresponding growth measures. For models presented for length and weight, the initial measure of size (starting point) was at 20 weeks of gestation (femur length and estimated fetal weight), and for BMI at 6 months of age. The models are adjusted for child's sex and age at insulin and C-peptide measurement. Circles, length; squares, weight; triangles, BMI; wks, weeks; mo, months

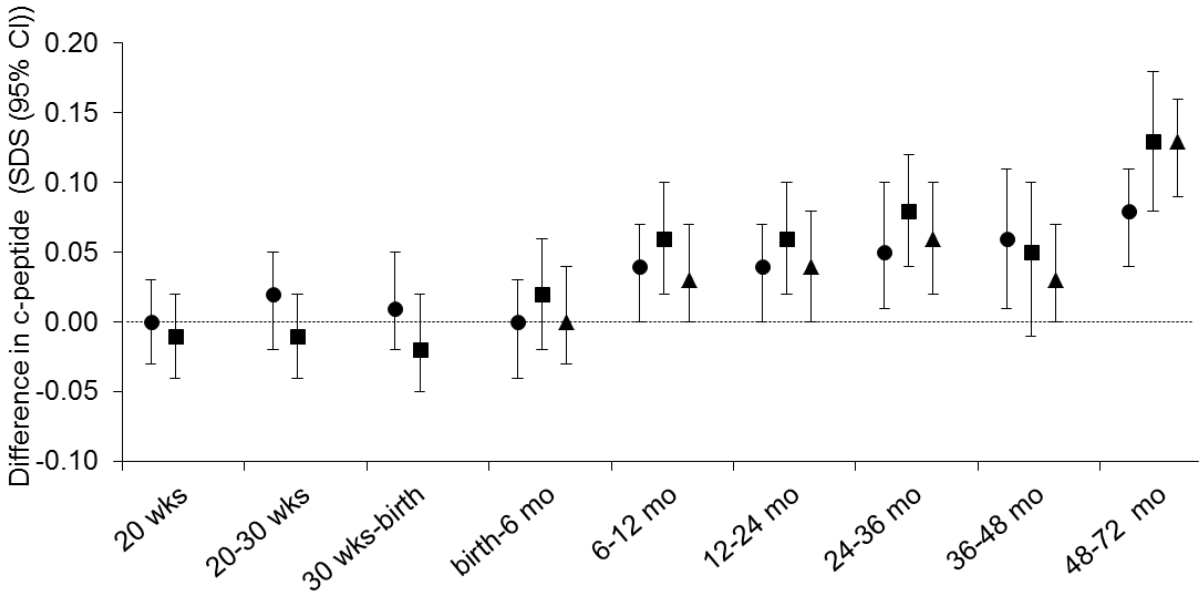

ESM Fig. 3 Associations of fetal and childhood growth conditional on prior measurements with childhood C-peptide levels (n=4303). Values are regression coefficients representing differences in childhood C-peptide SDS per standardised residual change in growth characteristic in each time interval. The standardised residuals were obtained from models in which the growth measures of interest were regressed on the prior corresponding growth measures. For models presented for length and weight, the initial measure of size (starting point) was at 20 weeks of gestation (femur length and estimated fetal weight), and for BMI at 6 months of age. The models are adjusted for child's sex, age at insulin and C-peptide measurement, maternal pre-pregnancy BMI, maternal age, parity, smoking during pregnancy, folic acid use, maternal education level, gestational diabetes, gestational hypertensive disorders, ethnicity of the child, gestational age at birth, breastfeeding, timing of introduction of solid foods and time watching television. Circles, length; squares, weight; triangles, BMI; wks, weeks; mo, months

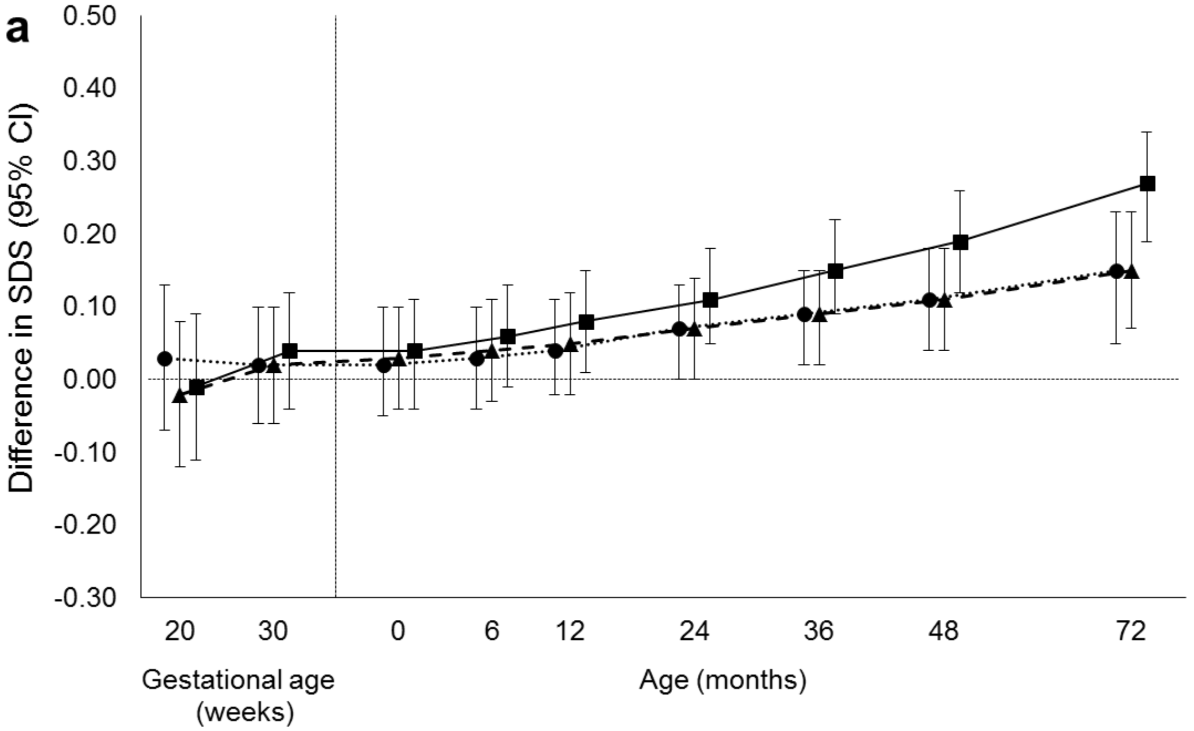
a
Difference in SDS (95% CI)
0.50
0.40
0.30
0.20
0.10
0.00
-0.10
-0.20
-0.30
20
30
0
6
12
24
36
48
72
Gestational age (weeks)
Age (months)

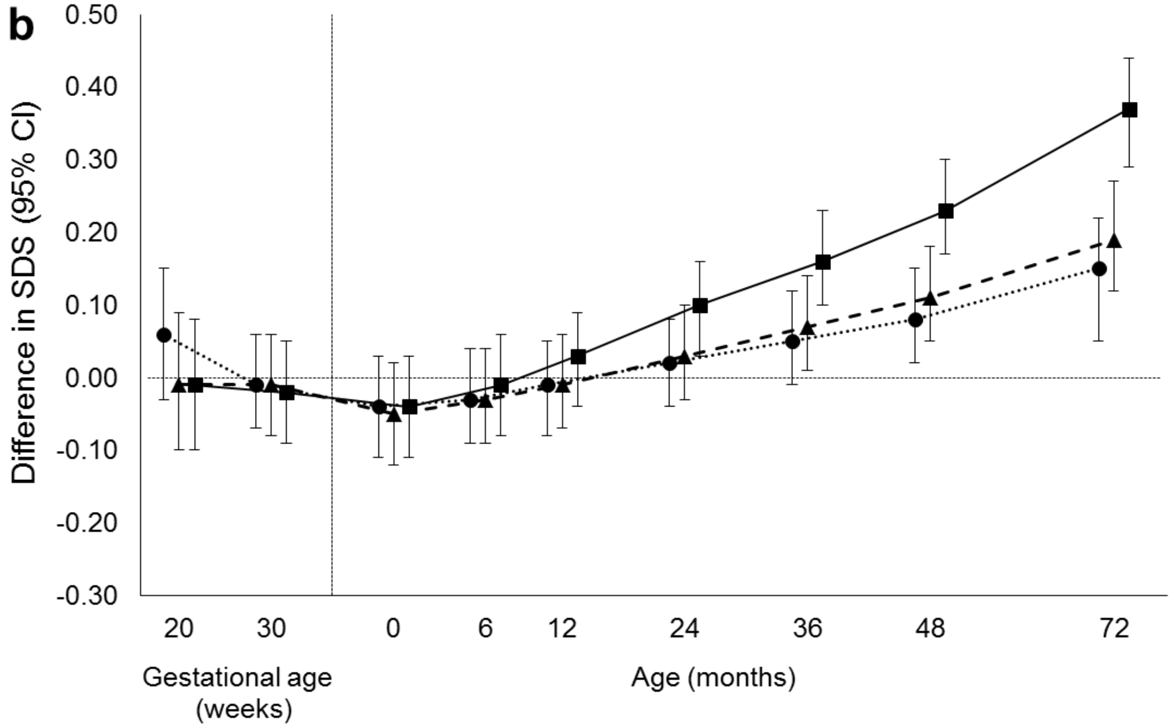
b
Difference in SDS (95% CI)
0.50
0.40
0.30
0.20
0.10
0.00
-0.10
-0.20
-0.30
20
30
0
6
12
24
36
48
72
Gestational age (weeks)
Age (months)

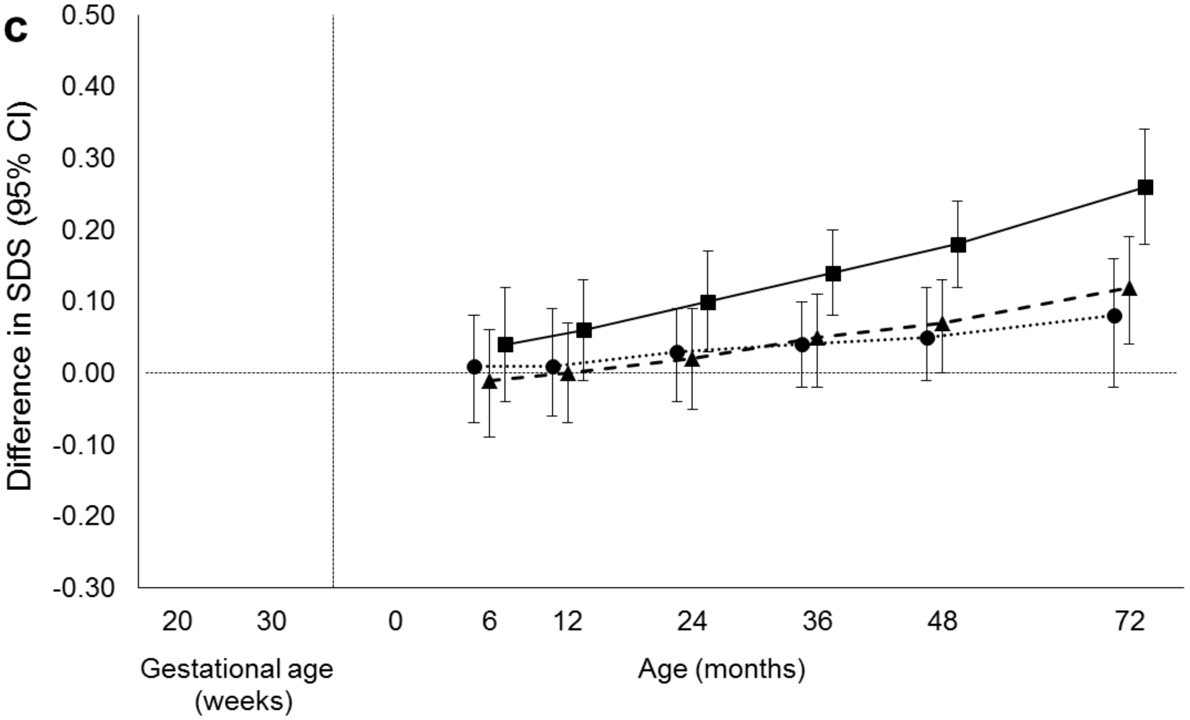

ESM Fig. 4 Fetal and childhood growth patterns according to C-peptide quartile (n=4303). Results are based on repeated linear regression models and reflect the differences in SDS of (a) length (based on 39,086 measurements), (b) weight (based on 42,692 measurements) and (c) BMI (based on 23,310 measurements) growth in children with C-peptide levels in the second, third and fourth quartiles (C-peptide levels 0.67-0.95, 0.96-1.28 and 1.29-3.69 nmol/l, respectively), compared with those with C-peptide levels in the first quartile (0.11-0.66 nmol/l). The reference value is an SDS of 0. The models were adjusted for child's sex, maternal pre-pregnancy BMI, maternal age, parity, smoking during pregnancy, folic acid use, maternal education level, gestational diabetes, gestational hypertensive disorders, ethnicity of the child, gestational age at birth, breastfeeding, timing of introduction of solid foods and time watching television. Circles/dotted line, second C-peptide quartile; triangles/dashed line, third C-peptide quartile; squares/solid line, fourth C-peptide quartile
